# Glomerular deposition of fibrinogen predicts good prognosis of IgA nephropathy: a single-center cohort study

**DOI:** 10.1007/s11255-023-03501-8

**Published:** 2023-02-14

**Authors:** Wei-guang Yang, Rong Zhu, Jian-nan Zheng, Jun-xiao Zhang, Nan Liu, Li Yao, Lin-lin Liu

**Affiliations:** Department of Nephrology, The First Affiliated Hospital of China Medical University, 155 Nan Jing North Street, He Ping District, Shen Yang, 110001 Liao Ning China

**Keywords:** IgA nephropathy, Fibrinogen deposition, Predictor, Prognosis

## Abstract

**Purpose:**

It has been proven that fibrinogen deposition exists in IgA nephropathy (IgAN), but its clinical significance has not been identified. We aim to investigate the clinical implication of fibrinogen deposition in evaluating the activity and prognosis of IgA nephropathy.

**Methods:**

In this cohort, 935 adult IgAN patients were divided into 3 groups according to the intensity of glomerular fibrinogen deposition. Primary outcome refers to a composite event of either a ≥ 50% reduction in eGFR or ESRD (eGFR < 15 ml/min/1.73m2, dialysis, or renal transplantation). Factors associated with fibrinogen deposition and prognosis were identified.

**Results:**

The results showed that the intensity of fibrinogen deposition was positively correlated with eGFR (*P* < 0.001), serum albumin (*P* = 0.041), and hemoglobin levels (*P* < 0.05), but negatively correlated with age (*P* = 0.04), serum fibrinogen levels (*P* < 0.001), serum C4 (*P* = 0.023), the proportion of patients with hypertension (*P* = 0.003), and the percentage of glomeruli sclerosis (*P* < 0.001). The prognostic analyses identified that fibrinogen deposition was an independent predictor for the progression of IgAN (*P* = 0.033).

**Conclusion:**

Our study indicated that the deposition of renal fibrinogen can predict the prognosis of IgAN with high reliability.

## Introduction

IgA nephropathy (IgAN) is the most common form of primary glomerulonephritis throughout the world [1, 2]. IgAN is a mesangial proliferative glomerulonephritis, characterized by the predominant deposition of IgA (mainly galactose-deficient IgA1) in mesangium. The clinical and histological manifestations of IgAN are highly variable, and the prognosis is diverse correspondingly. About one-third of IgAN patients develop end-stage renal disease within 20–30 years after the initial diagnosis [3]. The previous studies have identified some risk factors for the progression of IgAN, but it is still challenging on this issue [4].

Fibrinogen is a soluble 340-kD plasma glycoprotein predominantly synthesized in the liver by hepatocytes with the chief function of hemostasis. It is mainly involved in blood coagulation and considered as a key regulator of inflammation in disease [5, 6]. In the recent years, fibrinogen had attracted attention for its unique role in the pathogenesis of kidney disease, mainly on the podocytes [7–10]. Published studies showed that the levels of urinary fibrinogen were elevated in some chronic kidney diseases and acute kidney injuries [11, 12]. Recently, the significance of the deposition of fibrinogen in IgAN has attracted much attention [13]. However, the predictive value of the deposition of fibrinogen has been unclear until now. In the present study, we explored the role of the renal deposition of fibrinogen for evaluating the activity and predicting the prognosis of IgAN with the data of a large cohort.

## Materials and methods

### Study population and data extraction

The patients were recruited consecutively according to the following criteria: (1) the diagnosis of IgAN was based on renal biopsy in our department from January 2013 to December 2017, which showed mesangial expansion or proliferation on light microscopy and significant deposition of IgA in mesangium by immunofluorescence; (2) all the patients were adults older than 14; (3) IgAN was not secondary, such as Henoch–Schonlein purpura, systemic lupus erythematosus, liver disease, ankylosing spondylitis, psoriasis, etc.; (4) no corticosteroids and immunosuppressants were treated before the start of the present study, which was defined as the day of renal biopsy. The data of follow-up were collected until December 2019.

Besides, we gathered the following clinical data before renal biopsy: sex, age, history of hypertension, serum levels of fibrinogen, C3, C4, IgA, IgM, IgG, creatinine, and uric acid, 24-h urinary protein excretion (UPE), red blood cell counts of each high power field in urine, etc. In the period of follow-up, serum creatinine, 24-h UPE, blood pressure, and therapeutic regiments were collected. The Chronic Kidney Disease Epidemiology Collaboration (CKD-EPI) formula was used for calculating the estimated glomerular filtration rates (eGFRs) [14].

### Renal pathological evaluation and detection of renal deposition of fibrinogen

Adequate renal tissue was obtained by renal biopsy to evaluate renal pathological lesions (eight glomeruli or more in light microscopy, immunohistology, and electron microscopy examination). Two pathologists evaluated the pathological manifestations separately according to Oxford classifications [15, 16], including mesangial proliferation, segmental glomerulosclerosis, endocapillary hypercellularity, tubular atrophy/interstitial fibrosis and crescents (cellular or fibrocellular crescents). Besides, we calculated the ratios of global sclerosis.

The location of renal fibrinogen deposition was investigated by immunofluorescent staining. The polyclonal rabbit anti-human fibronectin antibodies (Dako, Denmark) diluted 1:50 in PBS were applied to the slides and incubated at 37℃ for 1 h. Then the slides were washed in PBS three times. Renal deposition of fibronectin by immunofluorescence was detected using an Olympus BX51 microscope.

The grades of fluorescence intensity were defined according to the following criteria: negative, no fluorescence under a low-power lens, little fluorescence under a high-power lens; 1 + , little fluorescence under a low-power lens, some under a high-power lens; 2 + , some fluorescence under a low-power lens, clear under a high-power lens.

### Treatment protocol

The patients were treated according to the following items [17]. The patients with hematuria and/or UPE < 1 g/24 h with normal renal function were administrated with non-immunosuppressive therapeutic regimen, including angiotensin-converting enzyme inhibitors (ACEIs) or angiotensin II receptor blockers (ARBs), fish oil, statins, and anti-platelets. The patients with proteinuria of  ≥ 1.0 g/24 h and pathological manifestations of cellular/fibrocellular crescents, moderate to severe mesangial proliferation and/or interstitial cell infiltration were treated with immunosuppressive regimens, including corticosteroids, cyclophosphamide, mycophenolate mofetil, leflunomide or tripterygium glycosides alone or in combination, and non-immunosuppressive therapy were also administrated in these patients if necessary.

### Endpoints and definitions

The present study specified a composite event of either a ≥ 50% reduction in eGFR or ESRD (eGFR < 15 ml/min/1.73 m^2^, dialysis, or renal transplantation) as the primary outcome [17]. Hypertension was defined if arterial blood pressures were at or above 140/90 mmHg twice or more on different days, or if the target levels of less than 140/90 mmHg were reached with the control of anti-hypertensive medications [17].

### Statistical analyses

As we described in the previous study [17]. Normally distributed continuous variables were expressed as mean with standard deviation (SD) and compared with the *T* test or the analysis of variance, whereas skewed distributed continuous variables were expressed as median with quartile range and compared with the nonparametric test. The categorical variables were expressed with absolute frequencies and percentages and analyzed with the Chi-square test. To identify the independent prognostic value of renal fibrinogen deposition, a Cox proportional hazards regression model was applied for univariable and multivariable analyses by the “Enter” method. The Kaplan–Meier survival analysis was applied to estimate the predictive significance of renal fibrinogen deposition for the prognosis. Two-tailed *P* values less than 0.05 was considered statistically significant. SPSS version 22.0 (SPSS, Inc., Chicago, IL, USA) was used for all the analytic procedures.

## Results

### Baseline characteristics of the study population

There were 935 adult patients with IgAN included (447males and 488 females). The age was 37.96 ± 12.71 years old. The mean values for eGFR and proteinuria level were 82.85 ± 33.93 ml/min/1.73 m^2^ and 2.04 ± 2.24 g/day, separately. The percentages of the patients with chronic tonsillitis, hypertension, and hyperuricemia at study initiation were 32.3%. 39.8%, and 43.2% separately. The immunosuppressants were not administered before study initiation. Finally, 861patients (92.09%) had complete follow-up information. On average, the follow-up lasted 47 ± 15 months. Finally, 115 patients (13.36%) were exposed to the composite events, of which 67 patients (7.78%) progressed toward ESRD, and 48 patients (5.57%) developed a ≥ 50% reduction in eGFR.

### Correlations between fibrinogen deposition and clinical parameters

Immunofluorescent staining with renal tissue showed no fibrinogen deposition in 482 patients, glomerular fibrinogen deposition with an intensity of “1 + ” in 328 patients, and an intensity of “2 + ” in 125 patients. The baseline clinical characteristics grouped by F0, F1, and F2 are shown in Table 1.

**Table 1:** Baseline characteristics of the patients grouped by F0, F1, and F2

	F0	F1	F2	*P* value
Number	482	328	125	–
Sex (male %)	47.10	48.78	48.00	0.894
Age	37 (29–47)	35 (28–46)	34 (26–44)	0.04
Hypertension (%)	54.79	50.46	39.52	0.003
Urinary protein excretion (g/d)	1.37 (0.58–2.78)	1.42 (0.63–2.63)	1.06 (0.55, 2.15)	0.079
eGFR (ml/min/1.73m^2^)	82.39 (50.34–109.48)	94.04 (59.28–114.26)	102.39 (77.80–121.69)	0.041
Hemoglobin (g/L)	129.82 ± 20.29	133.38 ± 19.79	137.23 ± 21.74	< 0.05
Serum fibrinogen (g/L)	3.77 (3.13–4.56)	3.65 (3.08–4.28)	3.33 (2.93–3.93)	< 0.001
Serum IgA (g/L)	3.23 (2.48–4.08)	3.24 (2.46–4.12)	3.31 (2.55–4.13)	0.863
Serum IgG (g/L)	11.10 (9.09–13.2)	11.00 (9.04–12.95)	11.3 (9.32–13.35)	0.828
Serum IgM (g/L)	1.05 (0.75–1.45)	1.08 (0.78–1.52)	1.05 (0.74–1.35)	0.897
Serum C3 (g/L)	1.00 (0.87–1.14)	1.01 (0.86–1.18)	0.98 (0.84–1.12)	0.967
Serum C4 (g/L)	0.24 (0.20–0.29)	0.24 (0.20–0.28)	0.22 (0.18–0.27)	0.023
Serum albumin (g/L)	37.20 (33.60–40.30)	37.80 (34.50–40.60)	38.40 (35.13–41.20)	0.041
M1(%)	80.08	86.89	75.20	0.006
E1(%)	22.20	23.17	29.60	0.217
S1(%)	67.43	64.94	66.40	0.763
T1 and T2 (%)	47.10	40.85	29.60	0.001
C1 and C2 (%)	47.30	47.56	45.60	0.929
Glomeruli with global sclerosis (%)	0.19 (0.06–0.42)	0.15 (0.06–0.31)	0.09 (0–0.24)	< 0.001

*UPE* urinary protein excretion, *eGFR* estimated glomerular filtration rate

**Fig. 1:**
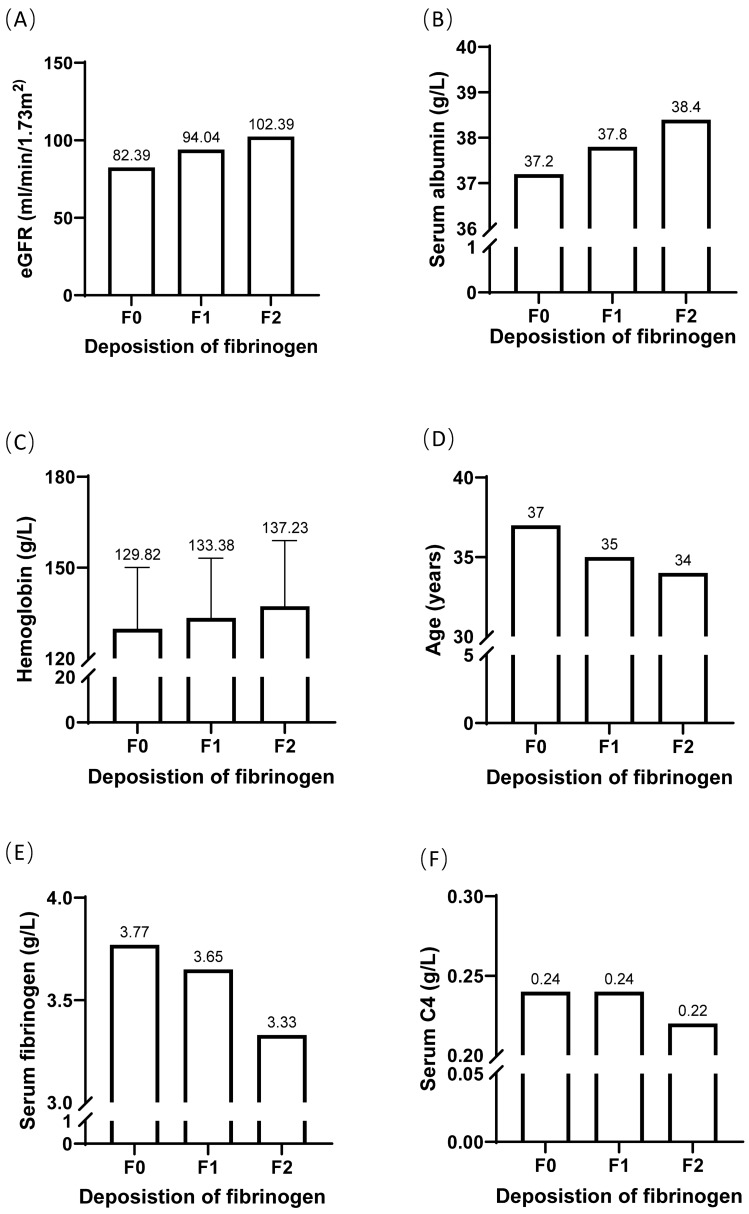
Comparison of clinical parameters among the patients with different degrees of fibrinogen deposition

The intensity of fibrinogen deposition was positively correlated with eGFR [F0, 82.39, IQR (50.34–109.48); F1, 94.04, IQR (59.28–114.26); F2, 102.39, IQR (77.80–121.69); *P* < 0.001], serum albumin [F0, 37.20, IQR (33.60–40.30); F1, 37.80, IQR (34.50–40.60); F2, 38.40, IQR (35.13–41.20); *P* = 0.041], and the levels of hemoglobin [F0, 129.82 ± 20.29; F1, 133.38 ± 19.79; F2, 137.23 ± 21.74; P01 = 0.015, P12 = 0.073, P02 < 0.001], but negatively correlated with age [F0, 37, IQR (29–47); F1, 35, IQR (28–46); F2, 34, IQR (26–44); *P* = 0.04], the levels of serum fibrinogen [F0, 3.77, IQR (3.13–4.56); F1, 3.65, IQR (3.08–4.28); F2, 3.33, IQR (2.93–3.93); *P* < 0.001], serum C4 [F0, 0.24, IQR (0.20–0.29); F1, 0.24, IQR (0.20–0.28); F2, 0.22, IQR (0.18–0.27); *P* = 0.023], and the ratios of patients with hypertension (F0, 54.79%; F1, 50.46%; F2, 39.52%, *P* = 0.003) (Fig. 1).

In addition, the levels of serum IgA increased in parallel to the intensity of fibrinogen deposition, but without statistical significance [F0, 3.23, IQR (2.48–4.08); F1, 3.24, IQR (2.46–4.12); F2, 3.31, IQR (2.55–4.13), *P* = 0.863]. The levels of urinary protein excretion in the patients with fibrinogen deposition were lower than those without fibrinogen deposition, but with no statistical significance [F0, 1.37, IQR (0.58–2.78); F1 & F2, 1.31, IQR (0.59–2.54), *P* = 0.449].

However, the condition of fibrinogen deposition showed no statistically significant correlation with serum levels of uric acid, IgG, IgM, and C3.

### Correlations between fibrinogen deposition and pathological parameters

**Fig. 2:**
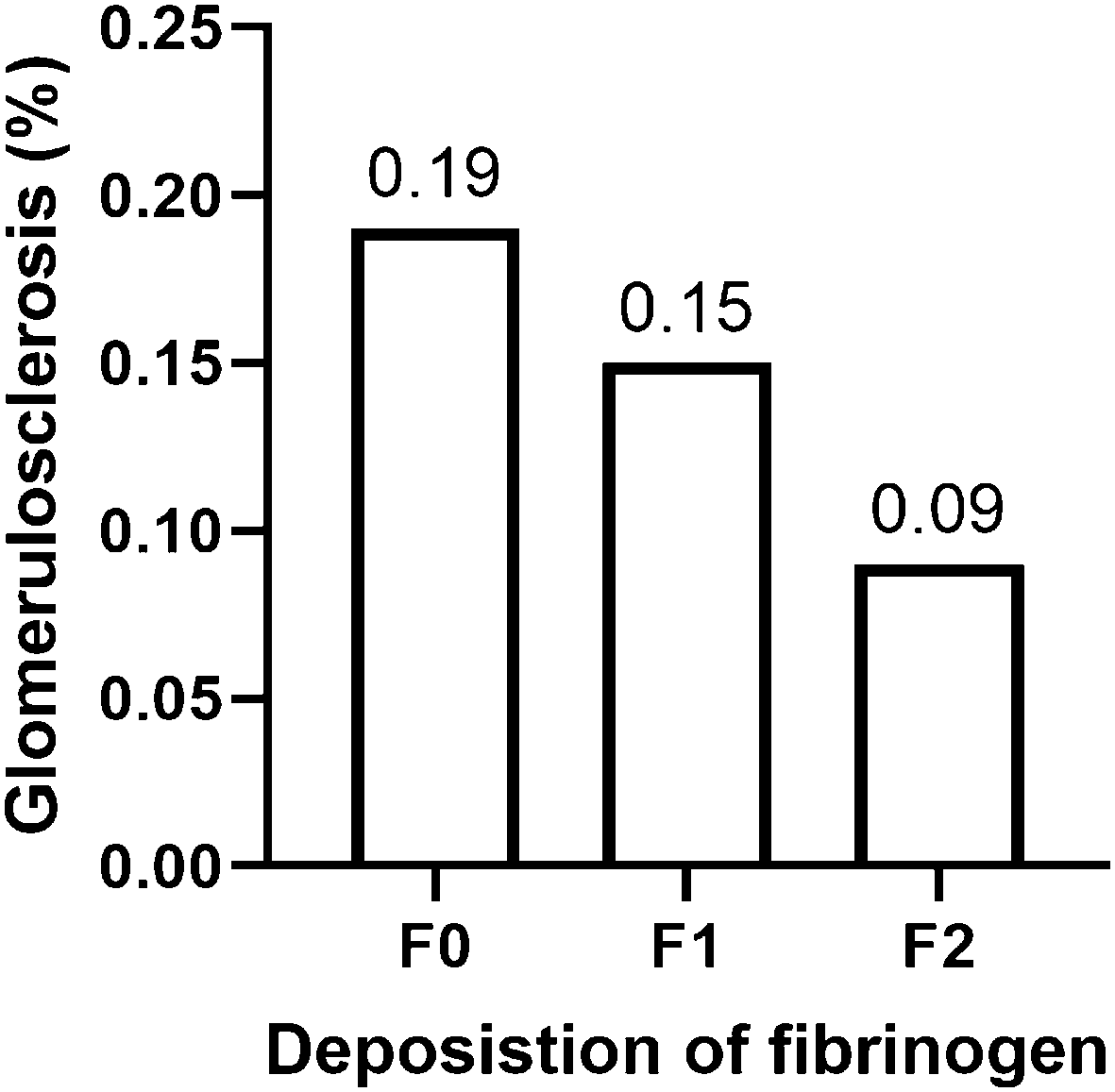
Comparison of the percentage of glomerulosclerosis among the patients with different degrees of fibrinogen deposition

The baseline pathological characteristics grouped by F0, F1, and F2 are shown in Table 1. The degree of fibrinogen deposition showed a significant negative relation to the percentage of glomeruli with global sclerosis [F0, 0.19, IQR (0.06–0.42); F1, 0.15, IQR (0.06–0.31); F2, 0.09, IQR (0–0.24); *P* < 0.001] (Fig. 2). The percentage of fibrinogen deposition decreased with the aggravation of tubulo-interstitial injury (54.01% vs 46.77% vs 32.37, P < 0.001). On the other hand, the fibrinogen deposition showed no statistically significant correlation with mesangial hypercellularity score (M), endocapillary hypercellularity (E), segmental sclerosis (S), and crescent formation (C).

### Correlation between renal fibrinogen deposition and prognosis

The patients followed up for more than 1 year were included for investigating the association of renal fibrinogen deposition with the prognosis.

Seventy-four patients (7.91%) were lost, and the mean follow-up period was 47 ± 15 months. Finally, 115 patients (13.36%) developed the study endpoint.

**Fig. 3:**
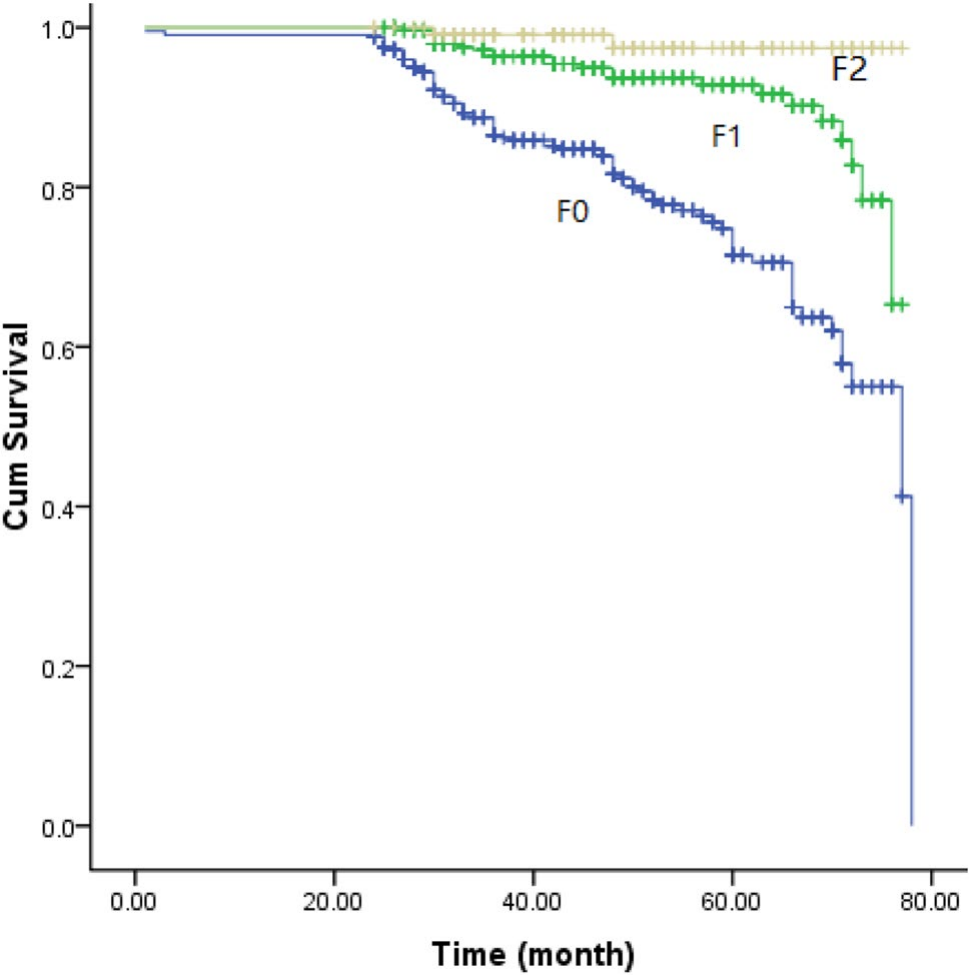
The Kaplan–Meier survival curves of patients with different degrees of fibrinogen deposition

The incidence rates of the composite endpoints decreased significantly with the increase of renal fibrinogen deposition (20.69% vs 7.42% vs 1.72%, *P* < 0.001). According to the univariable analysis, the following clinicopathological variables were identified as potential prognostic factors: sex, age, hypertension, proteinuria, renal function (i.e., eGFR), serum uric acid, IgA, C3, C4, and fibrinogen, administration with RASI, deposition of fibrinogen, M, S, T, C, and ratios of global sclerosis (Table 2). The multivariable analysis verified that glomerular deposition of fibrinogen was an independent predictor for the progression of IgAN (*P* = 0.033) (Table 2). The Kaplan–Meier survival curves showed no cross, indicating that the PH assumption was not violated, and revealed that glomerular deposition of fibrinogen well differentiated the prognosis in IgAN (Fig. 3).

**Table 2:** Univariate and multivariable Cox proportional hazards regression analysis of the data from the development cohort (the composite endpoint)

Predictors	Univariable analysis			Multivariable analysis		
	HR	95% CI	*P* value	HR	95% CI	*P* value
Sex			0.016			0.702
Male	1.000			1.000		
Female	0.630	0.432, 0.919		0.914	0.576, 1.449	0.702
Age	1.028	1.014, 1.042	< 0.001	1.011	0.993, 1.030	0.231
Hypertension			0.001			0.243
No	1.000			1.000		
Yes	1.936	1.329, 2.819		0.746	0.456, 1.220	0.243
Urinary protein excretion (g/d)	1.214	1.158, 1.273	< 0.001	1.051	0.962, 1.148	0.270
eGFR (ml/min/1.73 m^2^)	0.933	0.923, 0.943	< 0.001	0.960	0.945, 0.975	< 0.001
Serum fibrinogen (g/L)	1.480	1.318, 1.661	< 0.001	1.102	0.907, 1.338	0.328
Serum IgA (g/L)	0.879	0.749, 1.033	0.117	–	–	–
Serum C3 (g/L)	0.905	0.368, 2.228	0.828	–	–	–
Serum C4 (g/L)	200.597	31.263, 1287.098	< 0.001	6.059	0.442, 83.134	0.178
Serum uric acid (umol/L)	1.007	1.005, 1.008	< 0.001	1.001	0.999, 1.003	0.307
Urinary RBC (/HPF)	1.000	0.999, 1.001	0.979	–	–	–
Immunosuppressants			0.121	–	–	–
No	1.000					
Yes	1.393	0.917, 2.118				
RASI			< 0.001			0.019
No	1.000			1.000		
Yes	0.122	0.076, 0.197	< 0.001	0.482	0.262, 0.887	0.019
Mesangial hypercellularity			0.001			0.777
M0	1.000			1.000		
M1	3.543	1.722, 7.293	0.001	0.892	0.406, 1.960	0.777
Endocapillary hypercellularity			0.267	–	–	–
E0	1.000					
E1	0.768	0.482, 1.224	0.267			
Segmental sclerosis			< 0.001			0.803
S0	1.000			1.000		
S1	2.575	1.616, 4.104	< 0.001	0.933	0.544, 1.602	0.803
Tubular atrophy/interstitial fibrosis			< 0.001			0.042
T0	1.000			1.000		
T1	61.328	8.229, 457.032	< 0.001	12.655	1.645, 97.377	0.015
T2	371.493	51.764, 2666.079	< 0.001	14.382	1.808, 114.393	0.012
Crescents			< 0.001	–	–	–
C0	1.000		0.469			
C1	0.878	0.594, 1.300	0.517			
C2	1.320	0.694, 2.514	0.397			
Fibrinogen deposition			< 0.001			0.033
F0	1.000			1.000		
F1	0.295	0.186, 0.466	< 0.001	0.539	0.328, 0.885	0.015
F2	0.071	0.017, 0.287	< 0.001	0.439	0.104, 1.857	0.263
Glomerulosclerosis	275.621	134.802, 563.543	< 0.001	8.065	3.107, 20.933	< 0.001

*UPE* urinary protein excretion, *eGFR* estimated glomerular filtration rate, *RASI* renin–angiotensin system inhibitors, *RBC* red blood cell, *HPF* high power field

## Discussion

The present study with 935 IgAN patients showed that the deposition of fibrinogen in the mesangium was an important prognostic factor for IgAN. First, the intensity of fibrinogen deposition positively correlated with eGFR, serum albumin, and the levels of hemoglobin, but negatively correlated with age, the levels of serum fibrinogen, serum C4, and the ratios of patients with hypertension. Second, the degree of fibrinogen deposition showed a significant negative relation to the percentage of glomeruli with global sclerosis. Fibrinogen deposition decreased with the aggravation of tubulo-interstitial injury. Finally, fibrinogen deposition was identified as an independent predictor of IgAN progression in the prognostic analyses.

Accumulating evidence indicated that fibrinogen plays a critical role in acute inflammatory conditions. Some previous studies showed that serum fibrinogen levels increased in proinflammatory status and vascular inflammatory diseases [5, 18]. Another study put forward that renal fibrinogen deposition was associated with clinicopathological parameters [13]. However, no current study has investigated the prognostic value of renal fibrinogen deposition for IgAN. Younger patients, with less glomerulosclerosis and tubulo-interstitial injury, preserved their eGFR better, and lower proteinuria had a better outcome. Consequently, the facts in the present study identified the beneficial fibrinogen impact on the progression of glomerulosclerosis and IgAN-caused CKD. As a routine item in renal pathology, fibrinogen deposition is expected to be a convenient and effective predictor for IgAN.

According to our data, the deposition of fibrinogen was negatively correlated with the levels of serum fibrinogen. The discrepancy between them may originate from the fibrinogen captured by kidneys from serum. Besides, the mechanism of the good prediction of renal fibrinogen deposition in IgAN remains unclear. Some predictive mechanisms may start up after the fibrinogen deposited on the kidneys, which need to be investigated in future research.

In recent years, investigating useful predictors for the prognosis of IgAN has been a hot issue, particularly the biomarkers correlated with the pathogenesis [4, 19, 20]. On the basis of the useful biomarkers, some predictive models have been built [17, 21–23], which supply more accurate information on the prognosis of IgAN. The present study investigated the predictive value of the deposition of fibrinogen for IgAN, which may provide more clues for building the predictive models. Interestingly, most of the identified biomarkers are risk factors, which can usually predict the poor prognosis of IgAN. However, our study identified a predictive biomarker, which may indicate a good prognosis of IgAN.

The present study had some limitations. First, as a retrospective single-center study, our study could not exclude the limits of races, regions, or selection; and its external validity may be limited. Second, the discrepancy in therapeutic regimens may affect our conclusions. Last, we had not investigated the protective mechanisms of fibrinogen deposition for IgAN progression. Glomerulosclerosis may affect the deposition of fibrinogen, but we considered that it would not have an influence on the predictive significance of fibrinogen deposition in IgAN. We hope that we can explore the protective mechanisms of fibrinogen deposition in further research.

## Conclusion

The present study identified the deposition of fibrinogen as an independent predictor of the progression of IgAN. The deposition of fibrinogen was significantly correlated with the well-acknowledged clinicopathological prognostic factors, which indicated that it could be a good predictor of the prognosis of IgAN patients. However, further research is needed to investigate whether the deposition of fibrinogen can trigger some predictive mechanisms of IgAN.

## References

[1] Wyatt RJ, Julian BA (2013). Iga nephropathy. N Engl J Med.

[2] Lai KN, Tang SC, Schena FP (2016). Iga nephropathy. Nat Rev Dis Primers.

[3] D'Amico G (2004). Natural history of idiopathic iga nephropathy and factors predictive of disease outcome. Semin Nephrol.

[4] Suzuki H (2019). Biomarkers for Iga nephropathy on the basis of multi-hit pathogenesis. Clin Exp Nephrol.

[5] Adams RA, Schachtrup C, Davalos D, Tsigelny I, Akassoglou K (2007). Fibrinogen signal transduction as a mediator and therapeutic target in inflammation: lessons from multiple sclerosis. Curr Med Chem.

[6] Davalos D, Akassoglou K (2012). Fibrinogen as a key regulator of inflammation in disease. Semin Immunopathol.

[7] Sörensen I, Susnik N, Inhester T (2011). Fibrinogen, acting as a mitogen for tubulointerstitial fibroblasts, promotes renal fibrosis. Kidney Int.

[8] Craciun FL, Ajay AK, Hoffmann D (2014). Pharmacological and genetic depletion of fibrinogen protects from kidney fibrosis. Am J Physiol Renal Physiol.

[9] Motojima M, Matsusaka T, Kon V, Ichikawa I (2010). Fibrinogen that appears in bowman's space of proteinuric kidneys in vivo activates podocyte toll-like receptors 2 and 4 in vitro. Nephron Exp Nephrol.

[10] Wang H, Zheng C, Xu X (2018). Fibrinogen links podocyte injury with toll-like receptor 4 and is associated with disease activity in Fsgs patients. Nephrology.

[11] Wang H, Zheng C, Lu Y (2017). Urinary fibrinogen as a predictor of progression of Ckd. Clin J Am Soc Nephrol.

[12] Hoffmann D, Bijol V, Krishnamoorthy A (2012). Fibrinogen excretion in the urine and immunoreactivity in the kidney serves as a translational biomarker for acute kidney injury. Am J Pathol.

[13] Katafuchi R, Nagae H, Masutani K, Tsuruya K, Mitsuiki K (2019). Comprehensive evaluation of the significance of immunofluorescent findings on clinicopathological features in Iga nephropathy. Clin Exp Nephrol.

[14] Levey AS, Stevens LA, Schmid CH (2009). A new equation to estimate glomerular filtration rate. Ann Intern Med.

[15] Cattran DC, Coppo R, Cook HT (2009). The Oxford classification of Iga nephropathy: rationale, clinicopathological correlations, and classification. Kidney Int.

[16] Trimarchi H, Barratt J, Cattran DC (2017). Oxford classification of Iga nephropathy 2016: an update from the iga nephropathy classification working group. Kidney Int.

[17] Liu LL, Zhu LB, Zheng JN (2018). Development and assessment of a predictive nomogram for the progression of Iga nephropathy. Sci Rep.

[18] Zhang J, Chen C, Zhou Q (2017). Elevated serum fibrinogen level is an independent risk factor for Iga nephropathy. Oncotarget.

[19] Neprasova M, Maixnerova D, Novak J (2016). Toward noninvasive diagnosis of Iga nephropathy: a pilot urinary metabolomic and proteomic study. Dis Markers.

[20] Medjeral-Thomas NR, Lomax-Browne HJ, Beckwith H (2017). Circulating complement factor H-related proteins 1 and 5 correlate with disease activity in Iga nephropathy. Kidney Int.

[21] Barbour SJ, Coppo R, Zhang H (2019). Evaluating a new international risk-prediction tool in Iga nephropathy. JAMA Intern Med.

[22] Xie J, Lv J, Wang W (2018). Kidney failure risk prediction equations in Iga nephropathy: a multicenter risk assessment study in chinese patients. Am J Kidney Dis.

[23] Chen T, Li X, Li Y (2019). Prediction and risk stratification of kidney outcomes in Iga nephropathy. Am J Kidney Dis.

